# Partial starch substitution with resistant starch lowers postprandial glucose and glycaemic variability in people with type 2 diabetes

**DOI:** 10.1111/dme.70079

**Published:** 2025-07-09

**Authors:** Sineaid M. Collins, M. Bernadette Egan, Martin B. Whyte, M. Denise Robertson

**Affiliations:** ^1^ Faculty of Health and Medical Sciences University of Surrey Guildford UK

**Keywords:** cardio‐vascular disease, functional foods, HbA_1c_, postprandial glucose, resistant starch, starch substitution, type 2 diabetes

## Abstract

**Aims:**

Approximately 40% of the caloric intake of the UK diet consists of starch. Most of which is readily digestible, thereby raising blood glucose. However, resistant starch (RS) evades metabolism in the small intestine, and in healthy adults, partial substitution of the diet with RS lowers postprandial glycaemia. The effect of RS in adults with type 2 diabetes is unknown.

**Methods:**

We investigated the effect of substituting ~15% of dietary starch with RS on glycaemic measures in adults with type 2 diabetes in a controlled but free‐living setting. This was a single‐blinded, crossover design, comparing 4‐day RS and control diets. Proportions of resistant and digestible starch in identical food products were the only difference. IPro™2 continuous glucose monitors captured glycaemic excursions and glycaemic variability.

**Results:**

Twenty adults with type 2 diabetes, HbA_1c_ (52 ± 2 mmol/mol; 6.9 ± 0.3%), age 58 ± 11 years, were enrolled, with 95% completing arms. Mean amplitude of glycaemic excursion (MAGE) was lower over lunch with RS (0.94 mmol/L; *p* = 0.004), as was SD glucose (0.31 mmol/L; *p* = 0.027), and peak glucose (0.94 mmol/L; *p* = 0.028). RS prolonged the time it took glucose to peak by 18 min at lunch (*p* = 0.046) and 28 min at dinner (*p* = 0.002). Time in range (TIR; glucose 3.9–10.0 mmol/L) was 7.8% greater with RS (*p* = 0.021).

**Conclusion:**

Substituting a proportion of starch with RS lowers blood glucose without changing the sensory attributes of foods significantly. There is potential to develop a functional diet for adults with type 2 diabetes to aid glycaemic control.

**What's new?**

**What is already known?**

- Dietary approaches for the normalisation of PPG focus on low carbohydrate.
- Resistant starch passes (undigested) to the colon and thereby contributes little to postprandial glucose.

**What this study has found?**

- In a free‐living study of people with type 2 diabetes, substitution of 15% of dietary starch with resistant starch, improved measures of postprandial glucose, without changing the sensory attributes of foods.

**What are the implications of the study?**

- There is potential to develop a functional diet, using resistant starch, for adults with type 2 diabetes, to aid glycaemic control.

## 1 INTRODUCTION

The postprandial plasma glucose (PPG) response contributes significantly to glycated haemoglobin (HbA_1c_) in people with type 2 diabetes.^1^ Furthermore, PPG spikes are implicated in cardiovascular disease (CVD) pathogenesis by promoting oxidative stress in the vascular endothelium.^2^ As HbA_1c_ gets closer to the general target of 7% (53 mmol/mol), the contribution of PPG to HbA_1c_ is proportionally greater, making up to 70% of the total glycaemic contribution. Approximately 50% of those with type 2 diabetes fail to achieve HbA_1c_ of <7%,^3^ which highlights the difficulty in achieving optimal glycaemic control. PPG is, therefore, an important therapeutic target for people with type 2 diabetes.^4^ However, pharmacological treatments for PPG, such as sulfonylureas and insulin, can increase the risk of hypoglycaemia.^5^

Dietary approaches for the normalisation of PPG focus on low carbohydrate diets (LCDs). While efficacious in the short term, these diets can be challenging to maintain in the longer term.^6^ Increased protein and fat intake, combined with low starch/fibre ingestion associated with such diets, can lead to constipation and nausea,^7^ thereby lowering adherence. Conversely, diets high in RS can improve gastrointestinal health^8^ by elevating short‐chain fatty acid (SCFA) concentration.^9^ These metabolites, produced by commensal bacteria, signal via the gut–brain axis to elevate serotonin, gamma‐aminobutyric acid and GLP‐1 levels to improve satiety and mood.^10^ Greater satiety enhances insulin sensitivity through better appetite regulation, and reduced lipogenesis has also been reported.^11^ Starch, the primary carbohydrate source in UK diets, mainly consists of amylopectin, a non‐crystallising, water‐soluble, branched chain of repeating glucose units. Starch can be classified as resistant due to its botanical source, high amylose content or through cooking and processing.^12^ RS exhibits soluble and insoluble fibre characteristics, passing (undigested) to the colon and contributing little to PPG.^13^ Replacing ~14% of starch with RS significantly lowers PPG in healthy normoglycaemic adults and forms the basis of an existing health claim.^14^ This trial aimed to replace a proportion of dietary starch with RS and compare it with a control diet, comparing the effects of postprandial glycaemia in people with type 2 diabetes.

## 2 METHODS

### 2.1 Trial design

**FIGURE 1:**
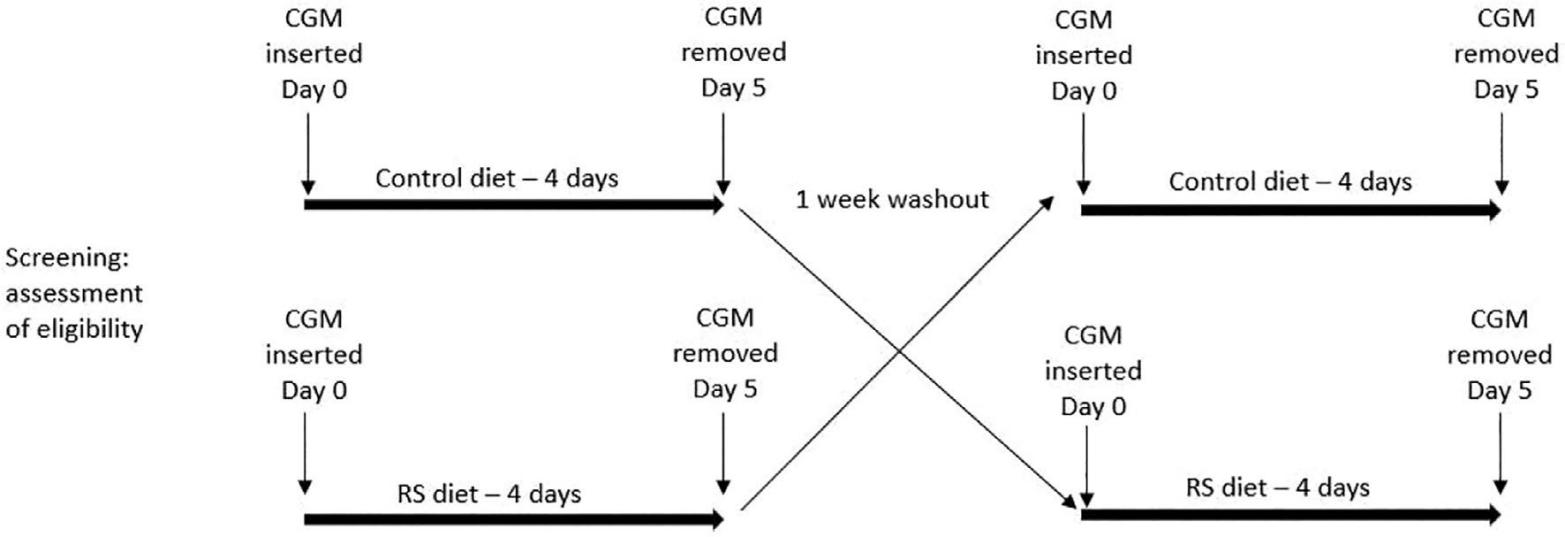
Crossover design of the study.

A single‐blinded crossover study to investigate the impact of replacing a proportion of starch with RS on interstitial glucose levels in people with type 2 diabetes (ISRCTN14165221) compared with a control diet. Each test diet was consumed over four consecutive days, separated by a minimum 1‐week washout (Figure 1). The trial was conducted between July 2019 and December 2020 at the Clinical Investigation Unit (CIU), Faculty of Health and Medical Sciences, University of Surrey, Guildford, UK. The authorised research ethics committee gave a favourable ethical opinion: South‐West—Cornwall & Plymouth Research Ethics Committee (18/SW/0204) UK. All participants gave written informed consent.

### 2.2 Masking and simple randomisation

Participants were randomly assigned to the order of intervention: control (A) or RS (B). Participants were blinded, as the control and RS diets were developed with comparable sensory attributes. Additionally, participants were blinded to their CGM data for the duration of the study.

### 2.3 Participants

Participants were recruited through primary care and identified by the Regional Clinical Research Network (CRN), a National Institute for Health Research (NIHR) division. Potential participants attended a short screening visit at the CIU to assess eligibility and obtain informed written consent. *Anthropometric measurements:* height by stadiometer (m), weight (kg) by Tanita multi‐frequency body composition analyser MC‐180MA (Tanita Inc., Japan) and a 5 mL blood sample were taken by venepuncture to measure baseline glycated haemoglobin (HbA_1c_) level. *Inclusion criteria:* type 2 diabetes >6 months duration, aged 18–70 years, access to a home kitchen, ability to understand English. *Exclusion criteria:* use of insulin and GLP‐1 analogues, history of gastric surgery or upper gastrointestinal disorders, HbA_1c_ >75 mmol/mol (9%), pregnancy/breast‐feeding, excess alcohol intake (>14 units per week), dose adjustment of any oral hypoglycaemic drug within the previous month and antibiotic use in the last 3 months. Using the Henry equation, we calculated individual participants' daily energy requirement, including variables of age, sex, BMI and physical activity. Diets were based on the Scientific Advisory Committee (SACN) UK daily adult recommendations of macronutrient intake: ~50% carbohydrate, ~20% protein and ~30% fat.^15^ Strenuous exercise was avoided on study days, and activity was reported.

### 2.4 Sensory testing of bespoke resistant starch food products

To increase the range of RS products in the test diet, bespoke crackers and bagels were produced using high‐amylose maize (HiMaize, Ingredion LLC, New Jersey, USA) in collaboration with Campden BRI (Chipping Campden, Gloucestershire, UK). Sensory evaluation was conducted during diet development using the International Standard Organisation methodology. *Triangle test*: to identify sensory differences between control and RS bagels and crackers, participants were randomly given three samples (two are the same, one is different). Sampling left to right, participants then identified the “odd one out” based on texture and taste differences to ensure no significant difference. *Preference test*: to determine whether participants preferred the control or RS crackers/bagels. *Ranking test*: to investigate taste intensity differences between control and RS crackers/bagels using a numbered line between 1 and 10. Participants marked on this line, one being ‘least intense’ and 10 being ‘most intense’.^16^

### 2.5 Interventions

#### 2.5.1 Diets

Both control and RS diets were comparable and matched for food volume and type, with an average macronutrient composition: 47.1% ± 0.2 carbohydrate, 33.2% ± 0.2 fat and 19.5% ± 0.2 protein (mean ± SD). Table 1 shows an example of a daily diet and illustrates the difference between diets related to the starch fraction and carbohydrate composition. There was an approximate 16% difference in RS levels compared to the control diet. All other food items were matched for food weight (Table 2). Study foods were a combination of commercially available and bespoke products. All meal items were individually pre‐weighed and labelled with simple, standardised preparation instructions to aid compliance. Participants were asked to consume breakfast, lunch and dinner on the same day on study days, using timing participants would habitually eat (Figure 1), with mealtime and any deviation recorded.

**TABLE 1:** An example of a daily diet consumed during the trial's control, and the RS arm illustrates the carbohydrate composition (g): starches, sugar and fibre. Using the Henry equation and Scientific Advisory Committee (SACN) to ensure daily recommendations of ~50% carbohydrate, ~20% protein and ~30% fat.

Starch‐based foods: control diet (337 g)						
Macro's (g)	Quick oats	Amioca bagel	Amioca cracker	White pasta	Banana chips	Total
Total grams	75	87	40	90	45	337
Total carb'	46.9	56.4	37.1	30.7	25.2	196.3
Starch	43.9	52.5	35.2	27.7	17.4	176.7
Sugar	0.74	2.34	1.24	1.53	5.6	11.3
Fibre	6.73	1.53	0.62	1.44	1.11	11.4
RS	2.05	1.53	0.62	1.44	1.11	6.7
Protein	9	7.8	3.2	5.4	1.3	32.7
Fat	6.1	1.6	2	0.8	11.3	22.7
Kcal	275	243	176	145	229	1068

Starch‐based foods: RS diet (337 g)						
Macro's (g)	Oats	RS bagel	RS cracker	RS pasta	Plantain chips	Total
Total grams	75	87	40	90	45	337
Total carb’	44.4	56.4	37.1	57.1	24.9	219.9
Starch	34.6	47.5	32.3	24.5	18.5	157.4
Sugar	0.82	2.34	1.24	0.01	2.56	6.9
Fibre	8.97	6.53	3.52	32.54	3.97	55.5
RS	7.8	6.53	3.52	32.54	3.97	54.3
Protein	7.7	8.2	3.2	7.4	0.9	33.7
Fat	5.2	1.7	2	9	10.3	21
Kcal	272	216	156	161	219	1024

Non‐starch‐based foods matched in weight, in both RS and control diets (898 g)										
Diet	Seeds	Berries	Egg	Sauce	Tuna	Mayo’	Cheese	Milk	Yoghurt	Dessert
Control (g)	12	25	56	150	120	30	40	200	124	140
RS (g)	12	25	56	150	120	30	40	200	124	140

**TABLE 2:** Daily percentage of RS (%) from carbohydrate in starch‐based foods, given in the example diet (337 g) in Table 1. The daily percentage of RS (%) from total daily grams of food (1235 g) from the example diet represents the daily starch‐based foods (337 g) and the nonstarch‐based foods (898 g).

	RS—starch‐based foods (337 g) (%)	RS—daily food intake (1235 g) (%)
RS diet	18	5
Control diet	2	1
Difference	16	4

#### 2.5.2 Trial visits

Participants visited the CIU on day 0 and day 5 of each diet. *Visit 1 (Day 0)*: The iPro™2 CGM system (Medtronic MiniMed, Northridge, CA) was fitted using an Enlite Serter (Medtronic MiniMed, Northridge, CA), with the Enlite sensor inserted subcutaneously 2 inches from the umbilicus. The CGM system was calibrated with four finger‐prick capillary blood glucose readings taken at fasting, pre‐meal and bedtime each day of the trial, using a factory‐calibrated glucometer (Sinocare Glucose Meter System, Changsha, China). This ensured that interstitial glucose readings from the sensor consistently matched capillary blood glucose readings. *Days 1–4*: The trial was free‐living, but participants consumed only the set meals provided. *Visit 2 (Day 5)*: Participants handed in completed dietary sheets, and the CGM system was removed.

### 2.6 Focus group

Following the trial, participants interested in the focus group were invited to participate in a short, semi‐structured, 20‐min interview by phone or Zoom to discuss their diet experience. The aim was to elicit participants' views on their experience of consuming the diet and what would facilitate the long‐term use of such a diet. All interviewees provided informed consent prior to the interview and agreed to the interviews being recorded for analysis purposes. Recordings were transcribed to a secure University server and used to identify the key points made by participants.

### 2.7 Outcomes

The primary outcome measure was the mean amplitude of glucose excursion (MAGE), measured 150 min from the start of each meal (breakfast, lunch and dinner), including mealtime and immediate postprandial periods. Secondary outcomes, measured for 180 min from the start of breakfast, lunch and dinner, were mean glucose (mmol/L), standard deviation (SD) of mean glucose (a marker of glucose variability), peak glucose (mmol/L); time to glucose peak (TTP) and time in range (TIR: 3.9–10.0 mmol/L) measured in minutes. Other (daily) outcome measures included mean glucose and SD of mean glucose (mmol/L), TIR, time above range (>10.0 mmol/L) measured as a percentage of time, and total area under the curve (AUC) (mmol/L/min). Participant check sheets corroborated meal start times from CGM data, and any significant deviation was excluded from the final analysis.

### 2.8 Statistical analysis

A sample size of 20 participants was calculated to provide 98% power to detect a difference of postprandial MAGE of 0.8 mmol/L with a 5% significance level, assuming a within‐person SD of mean blood glucose of 1.0 mmol/L.^17^ The FAO/WHO recommends testing a glycaemic index with 8–10 individuals.^18^ However, we increased the sample size here due to higher variability in the diabetic population. Seventeen participants completed the study with full adherence to the study protocol. Following a dietary run‐in on Day 1, continuous glucose monitoring (CGM) data from Days 2–4 were used to compare PPG and glucose variability between diets using a repeated measures one‐way ANOVA in SPSS v25 (IBM Corp, Illinois, U.S.A). Data are presented as mean ± SD. All data were checked for normalcy using Shapiro Wilk testing and subsequently analysed using one‐way ANOVA repeated measures, except non‐parametric data which include Time in Range (TIR), Time to Peak (TTP) at breakfast, lunch and dinner (mins), as well as daily measures of Time in Range (%) and time above range (%). These were analysed using *U* Mann–Whitney testing to determine if median scores were statistically different between control and RS diets.

## 3 RESULTS

**FIGURE 2:**
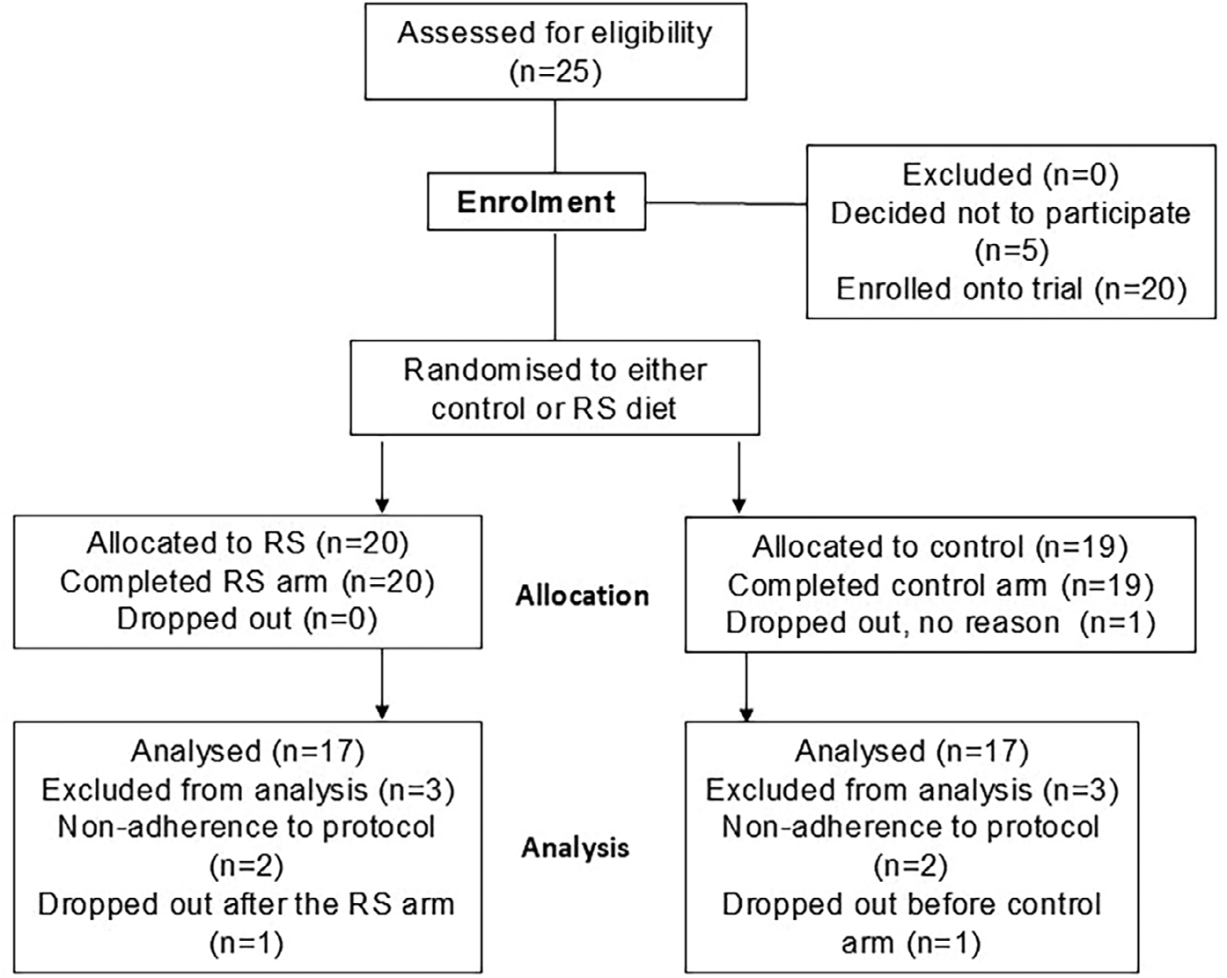
Participation flow through the study.

Twenty‐five of the 42 people with type 2 diabetes who responded to the trial invitation were screened and enrolled, with 19 fully completing the trial (Figure 2). Mean age was 58.2 years ±11, BMI 29.5 ± 6 kg/m^2^ and HbA_1c_ 52 ± 10 mmol/mol (6.9 ± 0.3%). The duration of type 2 diabetes was 9.0 ± 5 years. None smoked, and 59% were women. Oral hypoglycaemic medication was used in 55%. Statin therapy was taken by 53% of trial participants (Table 3).

**TABLE 3:** Baseline participant characteristics.

Characteristic	Mean ± SD (*n* = 17)
Age (years)	58 ± 11
Height (cm)	168 ± 12
Weight (kg)	84 ± 20
BMI (kg/m^2^)	29 ± 6
HbA1c (mmol/mol) (%)	52 ± 10 (6.9% ± 0.3)
Duration of type 2 diabetes (y)	9.0 ± 5
Smokers (%)	0
Men (%)	45

### 3.1 Sensory evaluation

Twenty‐three healthy people participated in the taste tests to ensure that control bagels and crackers (made with Amioca starch, NJ, USA) were palatable and indistinguishable from the RS bagels and crackers. Most participants (≥70%) could not distinguish between the control and RS bagels and crackers. There was no clear preference for either type of bagels (*p* = 0.210) or crackers (*p* = 0.415), and there was no significant taste difference between control and RS bagels (*p* = 0.741) or crackers (*p* = 0.559).

All measurements of daily and meal glycaemia and glycaemic variability are shown in Table 4.

**TABLE 4:** Glycaemic and variability measures obtained from CGM data during RS and control arms representing the mean of three consecutive days ± SD (*n* = 17). Glycaemic target range 3.9–10.0 mmol/L.

	Control diet	RS diet	Difference	(*p*)
*Meal: glucose variability*				
MAGE (mmol/L)				
Breakfast	3.9 ± 1.5	3.6 ± 1.4	0.4	0.360
Lunch	3.7 ± 1.2	2.8 ± 1.3	0.9	0.004
Dinner	3.3 ± 1.6	3.1 ± 1.4	0.2	0.679
Mean glucose SD (mmol/L)				
Breakfast	1.3 ± 0.50	1.2 ± 0.52	0.1	0.166
Lunch	1.2 ± 0.51	0.9 ± 0.54	0.3	0.027
Dinner	1.0 ± 0.54	1.0 ± 0.45	0.0	0.776
*Meal: glucose measurements*				
Peak glucose (mmol/L)				
Breakfast	11.1 ± 1.9	10.3 ± 0.8	0.8	0.311
Lunch	10.7 ± 1.8	9.8 ± 2	0.9	0.028
Dinner	9.8 ± 1.9	9.7 ± 2	0.1	0.851
Time to peak (mins)				
Breakfast	68 ± 28	72 ± 32	4	0.620
Lunch	90 ± 42	108 ± 44	18	0.046
Dinner	61 ± 24	89 ± 41	28	0.002
Time in range (mins)				
Breakfast	120 ± 51	144 ± 38	23	0.023
Lunch	121 ± 53	145 ± 54	24	0.005
Dinner	151 ± 6.3	145 ± 7.9	6	0.936
Mean glucose (mmol/L)				
Breakfast	8.9 ± 1.0	8.4 ± 0.9	0.5	0.041
Lunch	8.8 ± 0.9	8.1 ± 0.7	0.6	0.001
Dinner	8.4 ± 0.7	8.0 ± 0.9	0.3	ns
*Daily: glucose variability*				
Mean (mmol/L)	7.8 ± 0.2	7.6 ± 0.2	0.2	0.311
SD of mean (mmol/L)	1.3 ± 0.5	1.2 ± 0.1	0.4	0.166
Coefficient of variance (%)	17.8 ± 7.5	16.4 ± 6.2	1.5	0.299
% Time in range	83 ± 17	91 ± 10	8	0.021
% Time above range (>10 mmol)	15 ± 14	10 ± 11	5	0.020
Daily AUC (mmol/L/min)	11,360 ± 190	10,747 ± 270	613	0.066

*Note*: All data were analysed using one‐way ANOVA repeated measures, except for non‐parametric data; Time in range (TIR), Time to peak (TTP), measured in minutes, at breakfast, lunch and dinner, daily Time in range (%) and Time above range (%). These were analysed using *U* Mann–Whitney testing to compare differences in median scores.

### 3.2 Meal interstitial glucose variability

*MAGE:* Over the lunch period, a lower mean MAGE was observed following the RS diet (2.8 ± 1.3 mmol/L) compared to the control diet (3.7 ± 1.2 mmol/L), a difference of −0.9 mmol/L; *p* = 0.004. Furthermore, the SD of mean glucose with RS diet (0.9 ± 0.5 mmol/L) was lower than the control diet (1.2 ± 0.5 mmol/L), *p* = 0.027.

### 3.3 Meal glucose measurements

There was a post‐meal glucose‐lowering effect with the RS diet, primarily observed over the lunch period. *Peak Glucose:* Peak glucose was lower at lunch following consumption of the RS diet (9.8 ± 0.3 mmol/L) than control (10.7 ± 0.3 mmol/L), *p* = 0.028.

*TTP:* Glucose levels took 18 min longer to peak over the lunch period with the RS diet (108 ± 44 min) compared to the control (90 ± 42 min), *p* = 0046. At dinner, TTP was extended by 28 min with RS (89 ± 41 min) compared to the control diet (61 ± 24 min), *p* = 0.002.

**FIGURE 3:**
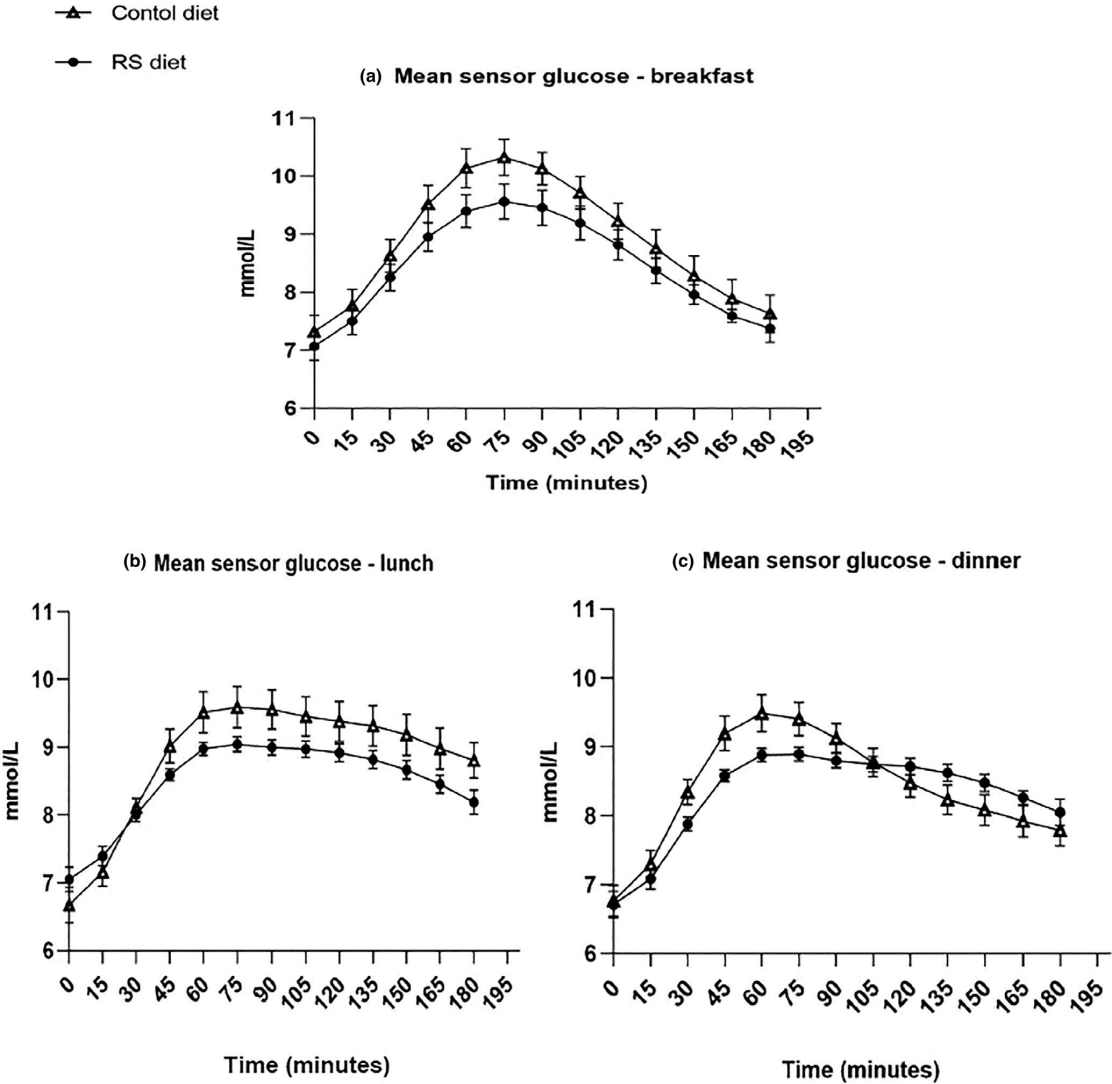
Mean glucose excursion following breakfast (panel a), lunch (panel b) and dinner (panel c) for *n* = 17 individuals with type 2 diabetes. Control starch (triangle), 15% substitution of control starch with resistant starch (circle).

*TIR:* Measured over the 180 min from the start of all meals, TIR was longer over the breakfast period with RS (144 ± 38 min) than control (120 ± 51 min), *p* = 0.02. Over the lunch period, a similar effect was observed: RS diet 145 ± 54 min versus control 121 ± 53 min, *p* = 0.005. *Mean glucose* was lower over breakfast with the RS diet (8.4 ± 0.9 mmol/L) versus control (8.9 ± 1.0 mmol/L), *p* = 0.041. Similarly, at lunchtime, the mean glucose was lower with the RS diet (8.1 ± 0.7 mmol/L) versus control (8.8 ± 0.9 mmol/L), *p* = 0.001 (Figure 3).

### 3.4 Daily (24‐h) glucose measurements

Daily mean measurements were calculated over 24 hours on replicate days 2, 3, and 4 from midnight to midnight. *Daily TIR:* The RS diet led to a 7.8% greater TIR than the control (*p* = 0.021) over 24 h. The time glucose spent above >10.0 mmol/L was 5.8% lower with the RS diet compared with control (*p* = 0.020). *Total AUC:* A mean reduction of 613 mmol/L/min in AUC with the RS diet compared to control (*p* = 0.066). *Daily mean glucose:* There was no significant difference in daily mean glucose between diets (0.3 mmol/L; *p* = 0.311). *Daily SD of mean glucose*: There was also no difference in the SD of daily mean glucose (0.1 mmol/L; *p* = 0.166) (Table 4).

### 3.5 Qualitative analysis

Ten participants were interviewed, six via Zoom and four by telephone. Overall, participants were very positive about participating in the trial and generally positive about the diet. Several people reported disliking some individual foods, that is, banana chips were deemed too sweet for some tastes. Lack of variety in the diet was mentioned, though there was an understanding that this was a pilot study. No one reported feeling hungry while consuming the diet. The key challenge reported was the timing of when to eat, and several participants found this restrictive, though all managed to adhere to the schedule. The short duration of the trial was viewed as helping to overcome this barrier. Participants were asked if long‐term consumption of such a diet would be viable. In principle, the majority felt this could be viable if more food were available. Adherence to such a diet would depend significantly on its benefits and if these outweighed the adjustments needed to one's lifestyle.

## 4 DISCUSSION

We have shown that in a free‐living environment, substituting a proportion of fully digestible starch with RS, compared to a control diet, reduces postprandial excursions and lowers glycaemic variability in people with type 2 diabetes. This is noteworthy because the average duration of type 2 diabetes was 9 years, and as a progressive disease, the longer you live with type 2 diabetes, the harder it becomes to manage.^19^ For the first time, we have been able to change the “functionality” of the widely consumed UK diet by replacing a small amount of a prevalent dietary component without changing sensory characteristics or eating behaviour. We lowered PPG by as much as 0.9 mmol/L over lunch. Lower PPG and glycaemic variability were observed in all five of the outcomes. The magnitude of such a change is clinically relevant. Madani et al. investigated factors predisposing to CVD in people with type 2 diabetes with adequately controlled glycaemia. They observed that a 1 mmol/L increment of PPG was associated with a 44% increase in the odds of CVD.^20^ Furthermore, monitored by CGM, sustained reduction in MAGE was associated with decreased circulating reactive oxygen species, a risk factor for CVD, in those with type 2 diabetes,^21^ independent of HbA_1c_ levels. Hence, the reduction in PPG fluctuation could be clinically significant if reproduced over extended periods.

Greater TIR is strongly associated with a risk reduction in the development or worsening of co‐morbidities^22^ and CVD.^23^ In our substitution study, we report a significant ~6% increase in daily TIR by changing one single component of the meal. Such a change in TIR would be expected to lead to a reduction in HbA_1c_ of up to 0.8% (DCCT units),^24^ broadly equivalent to outcomes by introducing an oral hypoglycaemic agent and can be achieved without loss of taste or variety in the diet.^25^

Overall, the RS diet led to a flattening of the postprandial glycaemic curve. Chronic RS consumption has beneficial effects on PPG, secondary to improvements in insulin resistance initiated by bacterial fermentation and thought to be independent of any acute meal‐based effects.^26^ However, direct substitution of starch with RS is novel, with the beneficial effects not previously elucidated in those with type 2 diabetes. The demonstration here that RS intake can acutely impact glycaemia is further evidence of RS's potential role in managing type 2 diabetes. PPG‐lowering effects were greatest over lunch, and given that meal composition has the greatest impact on postprandial glycaemia,^27^ differences in the macronutrient composition of the three meals may have led to a more pronounced effect at lunch. Blood glucose‐lowering effects from the RS pasta during the previous night or overnight oats at breakfast may be implicated in lower lunch PPG due to the well‐documented ‘second‐meal’ effects on glycaemia following RS consumption.^28^

Healthy adults may have greater glucose tolerance in the morning due to greater insulin sensitivity in the morning and improved beta‐cell effectiveness. In people with type 2 diabetes, this pattern is attenuated.^29^ Differences in PPG‐lowering effects may be due to meal composition and RS substitution levels. The RS Lunch had 33% more carbohydrates than breakfast or dinner and showed the greatest PPG‐lowering effect. The starch substitution with RS varied: breakfast (5.6%), lunch (12.4%) and dinner (29%). PPG‐lowering effects may, therefore, be subject to meal composition. We adhered to daily recommended nutrition guidelines, so it was not possible to match macronutrients at each meal, but dose–response effects are a worthy consideration for future research.

### 4.1 Strengths and weaknesses

People living with type 2 diabetes helped develop the diet, ensuring palatability. Sensory testing on the bespoke ensured that both diets had similar sensory properties. Weighing and labelling all food items aided adherence.

A limitation is that, as a free‐living study, it was not possible to fully control participants' diet and activity outside the study environment. Half the participants used oral hypoglycaemic drugs, but we ensured that there had been no dose change in the 3 months before the trial. Future work should explore microbial changes, as lower glycaemia may stem from RS fermentation by gut bacteria. This increases SCFAs, which serve as energy in the liver, reducing insulin production. SCFAs also inhibit cholesterol synthesis and enhance satiety, influencing glycaemia.^30^ This additive effect on glycaemia would depend on each participant's initial gut microbiota composition and diversity at the start of the trial.

In conclusion, substituting dietary starch with RS at ~15% significantly lowers daily PPG in those with type 2 diabetes. This demonstrates that modifying starch composition, a component of the UK diet, without changing the macronutrient proportions or eating behaviour may be advantageous in developing functional, palatable diets that support diabetes management.

## FUNDING INFORMATION

This project was funded by Diabetes UK.

## CONFLICT OF INTEREST STATEMENT

The authors declare that they have no conflict of interest.
